# Identification of factors during bronchoscopy that affect patient reluctance to undergo repeat examination: Questionnaire analysis after initial bronchoscopy

**DOI:** 10.1371/journal.pone.0208495

**Published:** 2018-12-06

**Authors:** Kazushi Fujimoto, Tsukasa Ishiwata, Hajime Kasai, Jiro Terada, Yu Shionoya, Jun Ikari, Naoko Kawata, Yuji Tada, Kenji Tsushima, Koichiro Tatsumi

**Affiliations:** 1 Department of Medicine, School of Medicine, Chiba University, Chiba, Japan; 2 Department of Respirology, Graduate School of Medicine, Chiba University, Chiba, Japan; 3 Health Professional Development Center, Chiba University Hospital, Chiba, Japan; 4 Department of Pulmonary Medicine, International University of Health and Welfare, School of Medicine, Chiba, Japan; University of Texas MD Anderson Cancer Center, UNITED STATES

## Abstract

**Background:**

Re-biopsy by bronchoscopy is an important part of treatment for patients with relapsed lung cancer; however, some patients refuse to undergo a re-examination due to discomfort during their first bronchoscopy. The aim of the present study was to determine factors causing discomfort during bronchoscopy and to identify the factors that affect patients’ reluctance to undergo repeat examinations via a questionnaire administered immediately after the initial bronchoscopy.

**Methods and findings:**

We evaluated 283 patients who underwent bronchoscopy at Chiba University Hospital between September 2015 and March 2017. Following bronchoscopy, the patients answered a questionnaire regarding the procedure. We identified patient characteristics and factors related to bronchoscopy that were associated with patients’ reluctance to undergo re-examination.

Two hundred nine patients were ultimately enrolled in the study. The factors affecting patient tolerance for re-examination were female sex (odds ratio [OR], 2.81; 95% confidence interval [CI], 1.43–5.53), discomfort during the examination (OR, 1.70; 95% CI, 1.13–2.56), and unexpectedness of discomfort during the examination (OR, 1.83; 95% CI, 1.19–2.81). Patients experienced discomfort most frequently owing to throat anesthesia (n = 50 [24%]).

**Conclusions:**

Comfort during bronchoscopy is an important factor influencing patient tolerance for re-examination. Expectations of discomfort during bronchoscopy, as indicated by instructions provided before examination, and throat anesthesia are also important factors. Detailed explanations about bronchoscopy and improvement of the methods of throat anesthesia could decrease patient discomfort and may help decrease patients’ reluctance to undergo re-examinations.

## Introduction

Repetitive biopsies have garnered considerable attention, as patients who undergo targeted molecular therapy for relapsing non-small-cell lung cancer often require follow-up biopsies. Follow-up bronchoscopies may also be required for patients using the novel cryo-transbronchial lung biopsy technique, which can provide important diagnostic indicators for interstitial lung diseases [1,2]. Because patients who experience discomfort may be less willing to consent to follow-up procedures, performing comfortable bronchoscopies has become increasingly important.

Lechtzin et al. investigated the satisfaction of patients with bronchoscopy and their willingness to return for a repeat bronchoscopy [3]. They found that better health status, dislike of scope insertion, better ratings of information quality, and better ratings of physician quality were all associated with the willingness of the patient to repeat the procedure. However, that report was published over fifteen years ago, and in the meantime, improvements in technology such as endobronchial ultrasound (EBUS) and virtual bronchoscopic navigation have led to changes in bronchoscopic procedures.

In pursuit of a clearer understanding of the current situation, the present study focused on factors that affect the reluctance of patients to be re-examined, using a questionnaire administered after the initial bronchoscopic procedure to evaluate their satisfaction with the bronchoscopy procedure. The aim of the study was to determine the factors that make for comfortable and safe bronchoscopies, and thereby to minimize the number of patients who are reluctant to undergo a re-examination.

## Material and methods

### Study design and materials

We retrospectively studied consecutive patients who underwent bronchoscopy at Chiba University Hospital between September 2015 and March 2017. After the patients recovered completely from the sedation during the bronchoscopies, they filled out a five-item questionnaire. Patients whose questionnaires were anonymous or included no responses, as well as patients who had previous experience of bronchoscopy, were excluded.

All study procedures involving human participants were approved by the Ethical Review Board of the Graduate School of Medicine of Chiba University (approval number 2584). The study protocol was conducted in accordance with the ethical principles of the 1964 Helsinki Declaration and subsequent amendments. Written informed consent was obtained from all participants. Furthermore, data was carefully managed with regards to respecting privacy, data protection, and civil rights.

### Bronchoscopy

All bronchoscopic procedures were performed using conventional flexible bronchoscopes (with one of the following bronchovideoscopes: BF-P260F, BF-P290, BF-260, BF-1T260, BF-1TQ290, BF-UC260-OL8; Olympus, Tokyo, Japan); the procedures were performed on either an inpatient or an outpatient basis. When outpatient doctors scheduled a bronchoscopy examination, they provided verbal and written explanations of the bronchoscopy procedure and obtained written consent from the patients. As pretreatment, hydroxyzine pamoate and atropine were administered by intramuscular injection, except in patients who had glaucoma or benign prostatic hyperplasia. A total of 5 ml of 2% lidocaine was sprayed into the throat with an ultrasonic nebulizer, and the same amount was then administered with a jet nebulizer. After performing throat anesthesia, the patients were asked to lay supine and were monitored every 5 minutes using electrocardiography, pulse oximetry, and blood pressure monitoring; expiratory CO_2_ was also monitored. Midazolam for sedation and/or pethidine for analgesia were administered intravenously during the bronchoscopy, and additional doses were used to maintain moderate sedation. The scope introduced through the mouth. When a bronchoscope was passed through the larynx and trachea, or when coughing occurred during the procedure, 2% lidocaine was administered through the bronchoscopy channel. If oxygen saturation decreased, supplemental oxygen was administered to maintain 90% oxygen saturation as measured by pulse oximetry. The bronchoscopies were performed by a pulmonologist who had over 5 years of experience with the procedure.

### Bronchoscopy questionnaire

Following the bronchoscopy, patients answered the following five questions: (1) “Did you feel that the bronchoscopy was uncomfortable?”; (2) “What was the cause of discomfort?”; (3) “How bad was the discomfort compared to your expectations before the examination?”; (4) “Do you remember the bronchoscopic examination?”; and (5) “How likely would you be to consent to a re-examination?”. The responses to questions 1, 3, and 5 were scored using a continuous scale ranging from 1 to 5, for which a visual analog scale was provided, with 1 signifying the most positive outcome and 5 signifying the most negative outcome. We defined the scores for questions 1, 3 and 5 as the “discomfort score”, “unexpected discomfort score”, and “reluctant score”, respectively. For question 2 (“What was the cause of discomfort?”), patients selected one of the following: throat anesthesia, cough, difficulty of respiration, pain, or other. For question 4 (“Do you remember the bronchoscopic examination?”), patients selected one of the following: remember, remember partly, remember nothing. The questionnaire was given to patients who underwent the examination on an in-patient basis, once it was confirmed that they were fully consciousness and a clear response was possible. The questionnaire was administered within twenty-four hours of the examination.

### Collection of associated data

The following data were also collected from the patients: indication for bronchoscopy (lung tumor, infection, diffuse lung disease, sarcoidosis, tuberculosis, other), position of lesion (central, peripheral, and diffuse), whether the bronchoscopy confirmed the diagnosis, duration of bronchoscopy, method of sedation (pethidine alone or midazolam and pethidine), type of bronchoscopic procedure (bronchoalveolar lavage, endobronchial ultrasound-guided transbronchial needle aspiration [EBUS-TBNA], transbronchial needle aspiration cytology, transbronchial biopsy, transbronchial lung biopsy [TBLB], other), and complications (none, pneumothorax, hemoptysis, hypoxia, pneumonia, other).

### Analysis of the questionnaire

**Fig 1:**
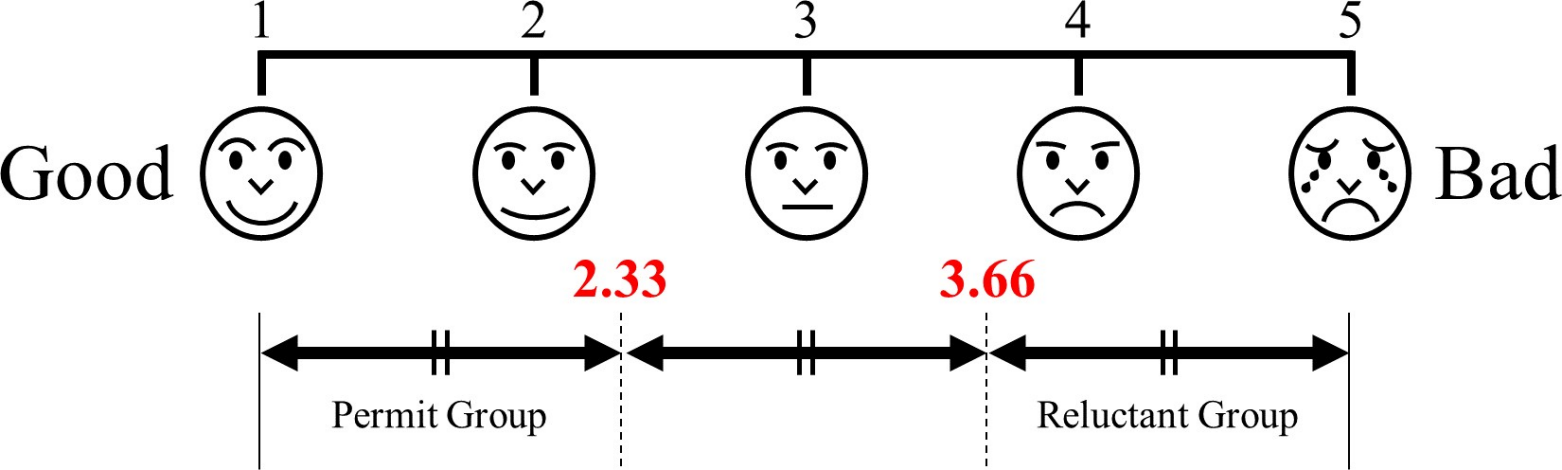
The visual analog scale of the bronchoscopy questionnaire and the group setting depends on the reluctant score.

The reluctant scores were compared with the associated data for all patients. Patients were then divided into 3 groups based on the reluctant score, which ranged in value from 1 to 5. Patients with reluctant scores less than 2.33 were categorized as the “Permit group,” and those with scores greater than 3.66 were categorized as the “Reluctant group” (Fig 1). Patients with scores between 2.33 and 3.66 were excluded because these patients did not clearly express either a willingness or reluctance to be re-examined. The parameters of the Permit group and Reluctant group were then compared.

Patients were then divided into 3 groups based on the reluctant score, which ranged in value from 1 to 5. Patients with reluctant scores less than 2.33 were categorized as the “Permit group” and those with scores greater than 3.66 were categorized as the “Reluctant group.”

### Statistical analysis

Continuous and categorical data were analyzed using the Mann–Whitney *U* test and the chi-squared test. Spearman’s correlation test was used to assess the correlation between each parameter and the reluctant score. Univariate and multivariate logistic regression analyses were used to identify the set of variables that would classify patients according to the reluctant score or other parameters. All results are expressed as the mean ± standard deviation unless otherwise indicated, and a P-value below 0.05 was considered statistically significant. All statistical analyses were performed using the JMP Pro 12.2.0 software program (SAS Institute Inc., Cary, NC, USA).

## Results

**Fig 2:**
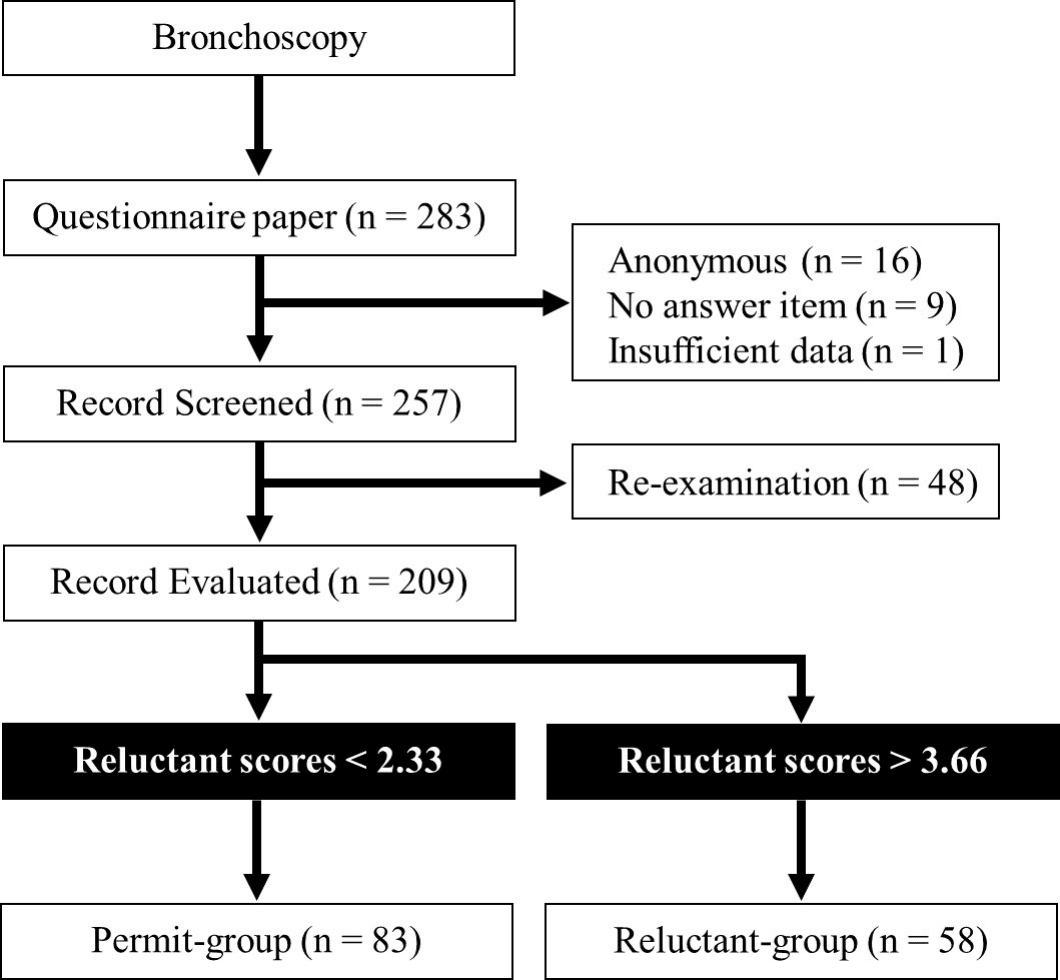
Flow diagram for the study.

A total of 283 patients who underwent bronchoscopy were included in the present study. Twenty-six patients were excluded because of anonymous questionnaires (n = 16), questionnaires with no answers (n = 9), or insufficient data (n = 1). An additional 48 patients were excluded from the study because they underwent a bronchoscopy previously (Fig 2). Finally, 209 patients were enrolled in the study.

Tables 1 and 2 summarize the patients’ baseline data and the correlations between the reluctant scores and the associated data. The reluctant score was significantly correlated with the discomfort score (r = 0.583, *p* < 0.001) and with the unexpected discomfort score (r = 0.565, *p* < 0.001). The factors that produced significantly higher reluctant scores were female sex (*p* < 0.001), sarcoidosis as the indication for bronchoscopy (*p* = 0.007), diffuse lesions (*p* = 0.041), pethidine alone for sedation (*p* = 0.006), TBLB (*p* = 0.019), and memory of the examination (*p* < 0.001). The rate of diagnosis confirmation, duration of bronchoscopy, and incidence of complications were not significantly correlated with the reluctant score. The proportion of patients describing throat anesthesia as a discomfort factor was greater among patients who had no memory of the procedure than among patients who remembered it (n = 26/87 [29.9%] vs. 24/122 [19.7%]; *p* = 0.101).

**Table 1:** Outline of bronchoscopy examination and correlations between reluctant score and each parameter (n = 209).

Parameter	Patients			Correlation coefficients	*p*-value
Age (y)	64.7 ± 12.4			-0.121	0.082
Sex, n (female/male)	89/120				**< 0.001**
Indication					
Lung tumor, n	122 (58%)				0.079
Infection, n	12 (6%)				0.729
Diffuse lung disease, n	53 (25%)				0.671
Sarcoidosis, n	19 (9%)				**0.007**
Tuberculosis, n	1 (0.5%)				0.726
Other, n	2 (1%)				
Position of the lesion					
Central, n	58 (28%)				0.896
Peripheral, n	80 (38%)				0.062
Diffuse, n	71 (34%)				**0.041**
Duration of examination (min)	25.8 ± 12.1			0.051	0.463
Sedation					
Pethidine, n	81 (39%)				**0.006**
Midazolam + Pethidine, n	128 (61%)				**0.006**
Midazolam, mg	1.6 ± 1.8			-0.132	0.056
Pethidine, mg	27.9 ± 7.8			0.043	0.533
Procedure					
TBAC, n	10 (5%)				0.687
TBLB, n	65 (31%)				**0.019**
TBB, n	101 (48%)				0.554
Brush, n	104 (50%)				0.050
EBUS-TBNA, n	42 (20%)				0.434
BAL, n	68 (33%)				0.061
BL, n	6 (3%)				0.129
Other, n	3 (1.4%)				
Number of diagnosis confirmation	147 (70%)				0.598
Incidence of complications	26 (12%)				0.385
Pneumothorax, n	1 (0.5%)				0.166
Hemoptysis, n	1 (0.5%)				0.286
Hypoxia, n	22 (11%)				0.269
Pneumonia, n	0 (0%)				
Other, n		2 (1%)			

BAL, bronchoalveolar lavage; BL, bronchial lavage; EBUS-TBNA, endobronchial ultrasound-guided transbronchial needle aspiration; TBAC, transbronchial needle aspiration cytology; TBB, transbronchial biopsy; TBLB, transbronchial lung biopsy. Data presented as mean ± standard deviation. Significant correlations printed in bold.

**Table 2:** Outline of answers to questions, and correlations between reluctant score and each parameter (n = 209).

Parameter	Patients	Correlation coefficients	*p*-value
1. Did you feel that the bronchoscopy was uncomfortable? (discomfort score)	2.5 ± 1.2	**0.583**	**< 0.001**
2. What was the cause of discomfort?			**< 0.001**
Throat anesthesia, n	50 (24%)		0.078
Cough, n	29 (14%)		**0.045**
Difficulty of respiration, n	22 (11%)		0.076
Pain, n	5 (2%)		0.160
Discomfort, n	8 (4%)		0.083
Other, n	2 (1%)		N/A
3. How bad was the discomfort compared to your expectations before the examination? (unexpected discomfort score)	2.2 ± 1.2	**0.565**	**< 0.001**
4. Do you remember the examination?			**< 0.001**
Remember, n	82 (39%)		**< 0.001**
Remember partly, n	40 (19%)		0.625
Remember nothing, n	87 (42%)		**< 0.001**
5. How likely would you be to consent to a re-examination? (reluctant score)	2.7 ± 1.4		N/A

Data presented as mean ± standard deviation. Significant correlations printed in bold.

### Comparison between the Permit group and the Reluctant group

Of the 209 patients in the present analysis, 83 were in the Permit group and 58 in the Reluctant group. Tables 3 and 4 summarize the characteristics of both groups. Female sex (36 [62%] vs. 24 [30%]; *p* < 0.001), sarcoidosis as the indication (10 [17%] vs. 3 [4%]; *p* = 0.006), and use of pethidine alone for sedation (27 [47%] vs. 24 [29%]; *p* = 0.032) were significantly more frequent in the Reluctant group than in the Permit group. In addition, the discomfort score (3.4 ± 1.2 vs. 1.8 ± 0.9; *p* < 0.001), unexpected discomfort score (3.1 ± 1.3 vs. 1.6 ± 0.8; *p* < 0.001), and degree of memory during the examination (“remember”, 32 [55%] vs. 20 [24%]; *p* < 0.001) were significantly higher in the Reluctant group than in the Permit group. Numerous patients from both groups experienced discomfort due to throat anesthesia (Reluctant group: n = 18 [31%], Permit-group: 14 [17%]). No significant differences were noted in the position of the lesion, procedure, rate of diagnosis confirmation, duration of bronchoscopy, and incidence of complications between the two groups.

**Table 3:** Outline of bronchoscopy examination in the Permit group and Reluctant group (n = 141).

Parameter	All (Permit + Reluctant Group)	Permit Group	Reluctant Group	*p*-value
Patient, n	141 (100%)	83 (59%)	58 (41%)	
Age (y)	64.2 ± 12.6	65.8 ± 12.0	62.0 ± 13.1	0.076
Sex, n (female/male)	60/81	24/59	36/22	**< 0.001**
Indication				
Lung tumor, n	88 (62%)	56 (67%)	32 (55%)	0.138
Infection, n	7 (5%)	5 (6%)	2 (3%)	0.488
Diffuse lung disease, n	31 (22%)	18 (22%)	13 (22%)	0.918
Sarcoidosis, n	13 (9%)	3 (4%)	10 (17%)	**0.006**
Tuberculosis, n	1 (1%)	0 (0%)	1 (2%)	0.130
Other, n	1 (1%)	1 (1%)	0 (0%)	
Position of the lesion				
Central, n	38 (27%)	23 (28%)	15 (26%)	0.808
Peripheral, n	59 (42%)	39 (47%)	20 (34%)	0.139
Diffuse, n	44 (31%)	21 (25%)	23 (40%)	0.070
Duration of examination (min)	26.2 ± 11.9	24.7 ± 9.8	28.3 ± 14.2	0.080
Sedation				
Pethidine, n	51 (36%)	24 (29%)	27 (47%)	**0.032**
Midazolam + Pethidine, n	90 (64%)	59 (71%)	31 (53%)	**0.032**
Midazolam, mg	1.6 ± 1.7	1.7 ± 1.5	1.5 ± 2.0	0.487
Pethidine, mg	28.1 ± 7.7	27.8 ± 7.9	28.5 ± 7.6	0.572
Procedure				
TBAC, n	7 (5%)	4 (5%)	3 (5%)	0.924
TBLB, n	41 (29%)	19 (23%)	22 (38%)	0.053
TBB, n	74 (52%)	45 (54%)	29 (50%)	0.622
Brush, n	79 (56%)	51 (61%)	28 (48%)	0.121
EBUS-TBNA, n	28 (20%)	14 (17%)	14 (24%)	0.287
BAL, n	39 (28%)	19 (23%)	20 (34%)	0.130
BL, n	5 (4%)	4 (5%)	1 (2%)	0.328
Other, n	2 (1%)	2 (2%)	0 (0%)	
Number of diagnosis confirmation	101 (72%)	58 (70%)	43 (74%)	0.581
Incidence of complications	18 (13%)	8 (10%)	10 (17%)	0.183
Pneumothorax, n	1 (1%)	1 (1%)	0 (0%)	0.402
Hemoptysis, n	1 (1%)	0 (0%)	1 (2%)	0.230
Hypoxia, n	14 (10%)	6 (7%)	8 (14%)	0.200
Pneumonia, n	0 (0%)	0 (0%)	0 (0%)	
Other, n	2 (1%)	1 (1%)	1 (2%)	

BAL, bronchoalveolar lavage; BL, bronchial lavage; EBUS-TBNA, endobronchial ultrasound-guided transbronchial needle aspiration; TBAC, transbronchial needle aspiration cytology; TBB, transbronchial biopsy; TBLB, transbronchial lung biopsy; Data presented as mean ± standard deviation. Significant correlations printed in bold.

**Table 4:** Outline of answers to questions in the Permit group and Reluctant group (n = 141).

Parameter	All (Permit + Reluctant Group)	Permit Group	Reluctant Group	*p*-value
Patient, n	141 (100%)	83 (59%)	58 (41%)	
1. Did you feel that the bronchoscopy was uncomfortable? (discomfort score)	2.5 ± 1.3	1.8 ± 0.9	3.4 ± 1.2	**< 0.001**
2. Why did you feel discomfort?				**< 0.001**
Throat anesthesia, n	32 (23%)	14 (17%)	18 (31%)	**0.048**
Cough, n	17 (12%)	6 (7%)	11 (19%)	**0.035**
Difficulty of respiration, n	14 (10%)	6 (7%)	8 (14%)	0.200
Pain, n	3 (2%)	0 (0%)	3 (5%)	**0.036**
Discomfort, n	6 (4%)	2 (2%)	4 (7%)	0.194
Other, n	2 (1%)	1 (1%)	1 (2%)	
3. How bad was the discomfort compared to your expectations before the examination? (unexpected discomfort score)	2.2 ± 1.3	1.6 ± 0.8	3.1 ± 1.3	**< 0.001**
4. Do you remember the examination?				**< 0.001**
Remember, n	52 (37%)	20 (24%)	32 (55%)	**< 0.001**
Remember partly, n	25 (18%)	15 (18%)	10 (17%)	0.899
Remember nothing, n	64 (45%)	48 (59%)	16 (28%)	**< 0.001**
5. How likely would you be to consent to a re-examination? (reluctant score)	2.7 ± 1.6	1.3 ± 0.4	4.5 ± 0.4	**< 0.001**

BAL, bronchoalveolar lavage; EBUS-TBNA, endobronchial ultrasound-guided transbronchial needle aspiration; TBAC, transbronchial needle aspiration cytology; TBB, transbronchial biopsy; TBLB, transbronchial lung biopsy; Data presented as mean ± standard deviation. Significant correlations printed in bold.

### Impacts on patient tolerance for re-examination

Table 5 presents the results of univariate and multivariate logistic regression analyses. After the reluctant score was converted into a binary variable using the median value as the splitting point, a univariate logistic regression analysis found associations with the reluctant score for female sex (odds ratio [OR]: 2.67, 95% confidence interval [CI]: 1.52–4.71, *p* < 0.001), sarcoidosis (OR: 3.05, 95% CI: 1.06–8.79, *p* = 0.028), diffuse lesions (OR: 2.06, 95% CI: 1.15–3.69, *p* = 0.014), TBLB (OR: 2.14, 95% CI: 1.17–3.90, *p* = 0.012), discomfort score (OR: 2.51, 95% CI: 1.87–3.37, *p* < 0.001), unexpected discomfort score (OR: 2.72, 95% CI: 1.97–3.77, *p* < 0.001) and memory of the examination (OR: 3.30, 95% CI: 1.86–5.88, *p* < 0.001). The multivariate logistic regression analysis, which included the variables that showed significance in the univariate analysis, revealed independent associations between the reluctant score and sex (OR: 2.81, 95% CI: 1.43–5.53, *p* = 0.002), discomfort score (OR: 1.70, 95% CI: 1.13–2.56, *p* = 0.011), and unexpected discomfort score (OR: 1.83, 95% CI: 1.19–2.81, *p* = 0.005).

**Table 5:** Logistic regression analysis of effect on reluctant score of each parameter (n = 209).

Parameter	Univariate Analysis			Multivariate Analysis		
	OR	95% CI	*p*-value	OR	95% CI	*p*-value
Age (per year)	0.99	0.97–1.01	0.317			
Sex, n (female/male)	2.67	1.52–4.71	**<0.001**	2.81	1.43–5.53	**0.002**
Time of examination (per minutes)	1.02	1.00–1.04	0.094			
Sarcoidosis, n	3.05	1.06–8.79	**0.028**	1.44	0.36–5.75	0.600
Diffuse lesions, n	2.06	1.15–3.69	**0.014**	1.83	0.76–4.40	0.177
Midazolam (per mg)	0.90	0.77–1.06	0.209			
Pethidine (per mg)	1.01	0.97–1.04	0.713			
TBLB, n	2.14	1.17–3.90	**0.012**	0.66	0.26–1.72	0.397
Discomfort score (per 1 point)	2.51	1.87–3.37	**<0.001**	1.70	1.13–2.56	**0.011**
Unexpected discomfort score (per 1 point)	2.72	1.97–3.77	**<0.001**	1.83	1.19–2.81	**0.005**
Memory during the examination (Remember/Never)	3.30	1.86–5.88	**<0.001**	1.33	0.65–2.75	0.438

CI, confidence interval; OR, odds ratio, Significant correlations printed in bold.

## Discussion

The present study focused on factors affecting discomfort during bronchoscopy procedures, which may influence patient reluctance to undergo a repeat examination after the initial examination. Female sex, sarcoidosis, diffuse lesions, anesthesia with pethidine alone, TBLB, discomfort during the examination, unexpected discomfort and memory of the examination all increased the patients’ reluctance to undergo a re-examination. Among these factors, female sex, discomfort during the examination, and unexpected discomfort had the strongest effects. Throat anesthesia was the most frequent cause of discomfort. Improvement is necessary, therefore, not only in the methods used for bronchoscopy itself but also for the preparations before the examination.

As expected, discomfort during bronchoscopy was found to be an important factor in patient reluctance to be re-examined. The method of sedation during bronchoscopy and the memory of the examination are presumed to have strong influences on patient perceptions of discomfort. The preferred drugs for sedation are combinations of the short-acting benzodiazepine midazolam with propofol or with other opiates such as fentanyl [4,5]. In the current study, patients who were administered both pethidine and midazolam remembered the procedure significantly less and had lower reluctant scores than patients who were administered pethidine alone (“remember nothing”: n = 80/128 [63%] vs. 7/81 [9%]; *p* < 0.001, 2.54 ± 1.34 vs. 3.03 ± 1.34; *p* = 0.007, respectively). In addition, there were no significant differences in rate of confirmation of the diagnosis, duration of the examination, or incidence of complications between the pethidine alone and combined pethidine and midazolam groups (confirmation: n = 94/128 [73%] vs. 53/81 [65%]; *p* = 0.217, 26.9 ± 12.1 min vs 24.0 ± 12.0 min; *p* = 0.059, complications: n = 15/128 [12%] vs. 11/81 [14%]; *p* = 0.691, respectively). A combination of pethidine and midazolam is one recommendation for more comfortable bronchoscopy.

Logistic regression analysis revealed that diffuse lesions as the position of lesions was associated with the reluctant score. In diseases with diffuse lesions, the proportions of diffuse lung disease and sarcoidosis were significantly higher than that of the other positions of lesions (diffuse lung disease: n = 41/71 [57.8%] vs. 12/138 [8.7%]; *p* < 0.001, sarcoidosis: n = 14/71 [19.7%] vs. 5/138 [3.6%]; *p* < 0.001, respectively), and that of lung tumor was significantly lower (n = 11/71 [15.5%] vs. 111/138 [80.4%]; *p* < 0.001). The duration of examination for diffuse lesions was 24.0 ± 12.1 min, and there was no significant difference in the duration of examination for diffuse lesions compared with the other positions of lesions (*p* = 0.138). The frequencies of TBLB and BAL were significantly higher among patients with diffuse lesions (n = 50/71 [70.4%] and n = 55/71 [77.5%], respectively; *p* < 0.001). In particular, the frequency of TBLB, BAL, and EBUS-TBNA was also higher among patients with sarcoidosis (n = 16/19 [84.2%], n = 18/19 [94.7%], n = 15/19 [80.0%], respectively). Therefore, the durations of examination for sarcoidosis tended to be longer (33.1 ± 2.6 min) among the patients with diffuse lesions. On the other hand, the proportion of patients who were administered a combination of midazolam and pethidine (n = 17/71 [23.9%] vs. 111/138 [80.4%]; *p* < 0.001), and the amount of midazolam (0.7 ± 0.2 mg; *p* < 0.001), was lower for diffuse lesions than for the other positions of lesions. Moreover, the proportion of patients who remembered the procedure was significantly higher among those with diffuse lesions (“remember” and “remember partly”, n = 57/71 [80.3%]; *p* < 0.001). However, the reason for a weaker sedation in case of diffuse lesions was not clear in this study. Thus, a high proportion of sarcoidosis and weak sedation could have affected the reluctant score in this study. As there can be variations in the composition of lung diseases and procedures for this type of bronchoscopy in different facilities, further studies with a larger number of cases with diffuse lesions are warranted to investigate this issue.

In addition to discomfort during the bronchoscopy, female sex and unexpected discomfort were other factors influencing patient reluctance to undergo re-examination. In the current study, the female patients were younger than the male patients (62.3 ± 13.0 years old vs. 66.5 ± 11.6 years old; *p* = 0.020). In addition, more female patients remembered the procedure than male patients (“remember”: n = 43 [48%] vs. 39 [32%]), although there was no significant difference between the sexes in the doses of midazolam and pethidine. Sex differences in pain have been a topic of increased interest in recent years [6]. Previous reports have found that postoperative and procedural pain may be more severe among women than men [6]. In addition, female patients more often fear bronchoscopy [7,8]. In a previous report investigating sigmoidoscopy for colorectal cancer screening, a higher percentage of women than men said that they would not repeat flexible sigmoidoscopy screening [9]. Further studies of the factors affecting female patients are needed to clarify why men and women are reluctant to be re-examined at different frequencies.

It is also important that patients be made aware of the potential discomfort of the examination, because the unexpected discomfort score was significantly lower in the Permit group than in the Reluctant group (1.6 ± 0.8 vs. 3.1 ± 1.3; *p* < 0.001). Although it is certainly necessary to minimize discomfort during bronchoscopy, it cannot be eliminated. In previous studies investigating factors that influence anxiety and satisfaction experienced by patients undergoing bronchoscopy, patients who showed willingness to be re-examined and underwent multiple bronchoscopies were on average more satisfied with their physicians’ explanations of the procedure [10,11]. Although patients fear a variety of things before bronchoscopy, such as pain, breathing difficulties, and oropharyngeal irritation, doctors tend to explain "why" but not "how" the procedure is performed [7]. Therefore, detailed explanation in advance of the entire process, what kind of pain to expect, and the methods of relieving discomfort could be helpful. Providing patients with detailed information about the sensations that are likely to be experienced could alleviate some of these common fears [7]. In our institution, we provide verbal and written explanations of bronchoscopy, and ask for consent beforehand. Dissemination of this type of information may be insufficient for patients to fully understand the procedure; therefore, improvements in the explanations, such as using more pictures or animations, might be helpful.

Throat anesthesia was the most frequently reported cause of discomfort related to bronchoscopy, in the full patient cohort as well as in the Permit group and Reluctant group (n = 50 [24%], 14 [17%], and 18 [31%], respectively). This suggests that there were patients who felt discomfort during the pre-bronchoscopy preparations, even if sedation during the bronchoscopy was sufficient. In our hospital, throat anesthesia was administered using a combination of an ultrasonic nebulizer (used by the patients on their own) and a jet nebulizer (used by the operating doctor), which was reported to be associated with the highest level of comfort during bronchoscopy [8]. However, in this study, the proportion of patients who described throat anesthesia as a discomfort factor was larger among those who had no vivid memory of the procedure than among those who remembered the procedure. While sufficient sedation during bronchoscopy may cause the patients to not remember the procedure, this lack of memory may cause the patients to perceive more discomfort arising from the throat anesthesia. It may even be possible to omit throat anesthesia if sufficient sedation is used during the bronchoscopy. Stolz et al. reported that additional nebulized lidocaine cannot be recommended for bronchoscopy performed under combined sedation [12]. It is necessary to pay attention to preparations for the examination such as sufficient explanations of the procedure and throat anesthesia as a pretreatment.

### Study limitations

The present study had three limitations. First, it was a single-center study with a small number of participants. Since various methods exist for sedation during bronchoscopy, throat anesthesia, and preliminary explanations of the examination, the small size of our study meant that only a limited set of these methods were compared. Therefore, further prospective multicenter studies involving larger patient populations are required to confirm these results. Second, not all patients who were reluctant to undergo a repeat examination after the first bronchoscopy would actually refuse re-examination. Willingness to be re-examined is affected by various factors such as the explanation given to the patients about the re-examination and the availability of treatment choice. However, to encourage patients to more readily accept re-examination, it is necessary to conduct as comfortable and safe examinations as possible. Therefore, we believe that the findings of this study will be useful for improving current examination practice. Third, recall bias could not be excluded in this study. This was an unavoidable problem due to the characteristics of our study design. Patients who might cause this bias could tend to feel more discomfort for bronchoscopy and, therefore, be more likely to remember the discomfort and be less willing to accept re-examination.

## Conclusions

In conclusion, comfort during bronchoscopy is an important factor that may influence patients’ reluctance to undergo a repeat examination after initial bronchoscopy. Expectations of discomfort, related to explanations provided to the patients before examination and throat anesthesia, are also essential factors. In addition to improvements in bronchoscopy devices and methods, and sedation during bronchoscopy, attention must be paid to the preparations for bronchoscopy, such as detailed explanations and sufficient throat anesthesia, if the goal is to increase the frequency of patients consenting to further bronchoscopy procedures.
